# Evaluating the impact of Age-Adjusted Charlson Comorbidity Index on in-hospital complications in patients with femoral fracture: a retrospective cohort analysis from the MIMIC-IV 2.2 database

**DOI:** 10.3389/fmed.2025.1606744

**Published:** 2025-05-27

**Authors:** Ping Xu, Liping Wang

**Affiliations:** Department of Osteology, Shaoxing Second Hospital, Shaoxing, Zhejiang, China

**Keywords:** Age-Adjusted Charlson Comorbidity Index, femoral fracture, deep venous thrombosis, pressure ulcers, delirium

## Abstract

**Background:**

Femoral fractures in hospitalized patients often lead to limited mobility, increasing the risk of complications like deep venous thrombosis (DVT), pressure ulcers, and delirium. These risks are particularly pronounced in elderly patients with multiple comorbidities. Monitoring such patients using reliable indices like the Age-Adjusted Charlson Comorbidity Index (ACCI) can help in early identification and management of these risks. This study investigates the association between ACCI scores and the incidence of in-hospital complications DVT, pressure ulcers, delirium in patients with femoral fractures.

**Methods:**

Using the MIMIC-IV 2.2 database, we extracted data for 4,134 patients diagnosed with femoral fractures after applying exclusion criteria such as repeated admissions, age under 18, and significant missing data. ACCI and other demographic and clinical data were used in logistic regression models and restricted cubic spline (RCS) analyses to assess the relationship between ACCI and complication risks.

**Results:**

A nonlinear association between ACCI and complication risk was identified, with significant risk increases beyond an ACCI of 5. Patients were divided into low-ACCI (≤5) and high-ACCI (>5) groups. High-ACCI patients had significantly greater risks of developing DVT (OR = 2.151), pressure ulcers (OR = 2.168), and delirium (OR = 1.791), compared to low-ACCI patients, indicating ACCI’s effectiveness in predicting these complications.

**Conclusion:**

ACCI is a valuable tool for predicting the risk of in-hospital complications among femoral fracture patients, facilitating targeted interventions and improved patient management.

## 1 Background

The global aging population presents significant challenges to healthcare systems, particularly concerning the management of age-related conditions such as femoral fractures (1). As the demographic of older adults continues to expand, the incidence of femoral fractures, particularly in individuals aged 65 and over, has risen sharply (2–4). The World Health Organization (WHO) has reported that the global burden of hip fractures is expected to increase, with projections suggesting that by 2050, the number of hip fractures could reach 6.3 million annually worldwide (5). This increase can be attributed to factors such as increased life expectancy, higher rates of osteoporosis, and a greater prevalence of falls among the elderly. Consequently, understanding the multifaceted implications of femoral fractures in older adults is paramount in developing effective treatment and management strategies.

In this context, the Age-Adjusted Charlson Comorbidity Index (ACCI) emerges as a crucial tool for assessing comorbidity in elderly patients (6–8). The ACCI is an adaptation of the original Charlson Comorbidity Index (CCI) (9), which was designed to predict mortality based on the presence of various comorbid conditions. By incorporating age as a factor, the ACCI provides a more nuanced understanding of the health status of older patients, reflecting the increased risk associated with advancing age (8). The ACCI has been validated in numerous studies, demonstrating its predictive validity for outcomes such as post-operative complications and mortality (5). This index is particularly relevant in the context of femoral fractures, as these patients often present with multiple comorbidities that can complicate their treatment and recovery (7).

The significance of investigating the relationship between ACCI and complications such as deep vein thrombosis (DVT), pressure ulcers, and delirium in elderly patients with femoral fractures cannot be overstated. DVT is a common complication following orthopedic surgeries, particularly in the elderly, due to factors such as immobility and underlying vascular disease. Pressure ulcers also pose a significant risk in this population, particularly among those with limited mobility during recovery. Delirium, characterized by acute confusion and cognitive impairment, is another prevalent issue in older adults, particularly in the postoperative setting. Understanding how the ACCI correlates with these complications can guide clinicians in risk stratification and management, ultimately improving patient outcomes. This study aims to explore the associations between ACCI and the aforementioned complications in elderly patients with femoral fractures, highlighting the importance of comprehensive assessment and individualized care strategies in this vulnerable population.

## 2 Patients and methods

### 2.1 Study design and data source

This study employed a retrospective cohort design utilizing the Medical Information Mart for Intensive Care IV (MIMIC−IV) version 2.2 database. The MIMIC-IV database is a freely accessible, extensive electronic health record repository that contains detailed clinical data from patients admitted to the intensive care unit (ICU) at the Beth Israel Deaconess Medical Center in Boston, Massachusetts, from 2008 to 2019. The database includes a wide array of patient information, including demographics, vital signs, laboratory test results, medications, and clinical outcomes, allowing for comprehensive analyses of patient cohorts. The use of the MIMIC-IV database was approved by the Institutional Review Board IRB at the Massachusetts Institute of Technology MIT, and all patient data were de-identified to protect patient privacy.

### 2.2 Inclusion criteria and exclusion criteria

**Figure 1:**
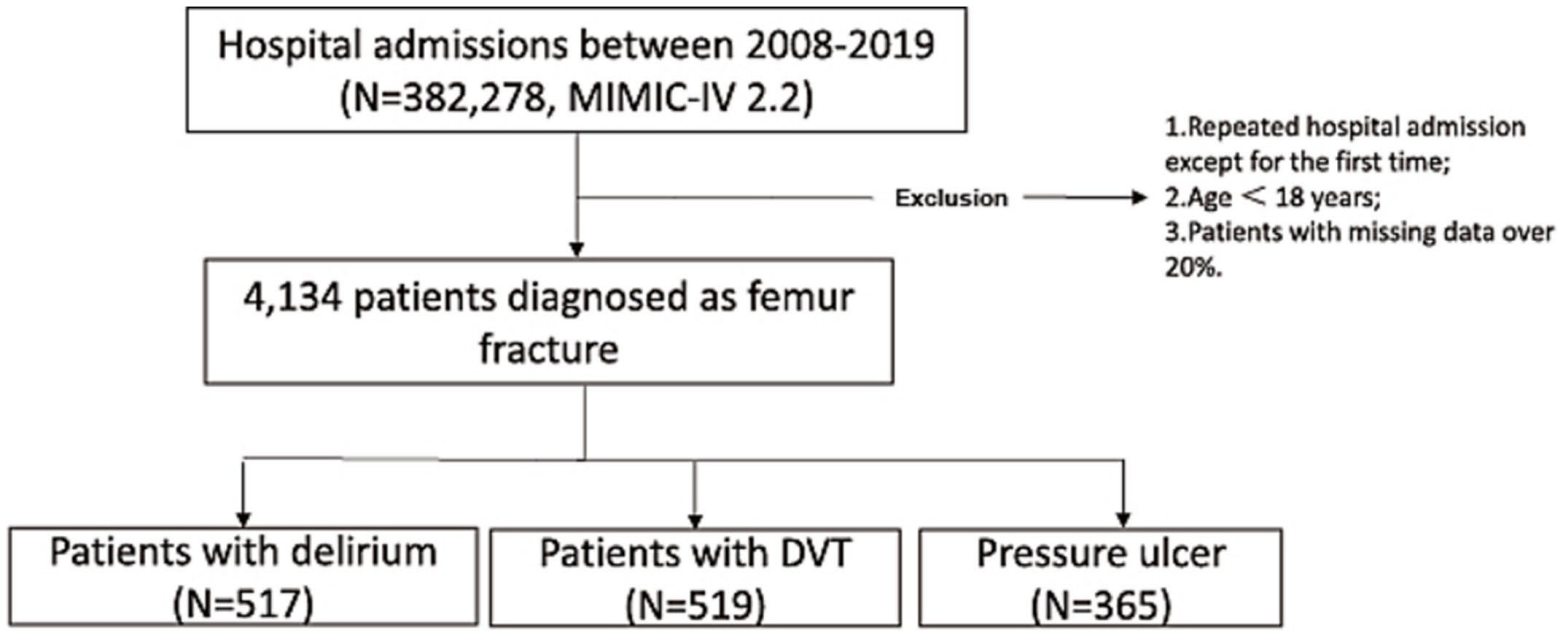
Screening flow diagram of patients with femoral fracture (MIMIC−IV, Medical Information Mart for Intensive Care IV; DVT, deep venous thrombosis).

Adult patients age ≥18 years admitted with a primary diagnosis of femoral fracture (all patients diagnosed with ‘femoral fracture’ upon admission, with the relevant ICD codes used as the basis for data extraction). Repeated hospital admissions, retaining only the first admission for each patient to avoid duplicity. The exclusion criteria will include: those with more than 20% missing data in key variables, ensuring data integrity and reliability for analysis. Screening flow diagram of patients with femoral fracture was described in Figure 1.

### 2.3 ACCI

The ACCI was calculated for each patient, incorporating the presence of various comorbid conditions such as dementia, myocardial infarction, congestive heart failure, peptic ulcer disease, cerebrovascular disease, rheumatic disease, mild liver disease, diabetes, paraplegia, renal disease, malignant cancer, AIDS, metastatic solid tumor, diabetes with complications, and connective tissue disease. The index also adjusted for age, providing a comprehensive assessment of the patient’s overall health status. Time-Adjusted Charlson Comorbidity Index: A Comprehensive Overview of ACCI was described in Table 1.

**Table 1:** Aged-adjusted Charlson comorbidity index: a comprehensive overview.

Comorbidity	Weight
Myocardial infarction	1
Congestive heart failure	1
Peripheral vascular disease	1
Cerebrovascular disease (stroke)	1
Dementia	1
Chronic pulmonary disease	1
Rheumatologic disease	1
Peptic ulcer disease	1
Mild liver disease	1
Diabetes without complications	1
Diabetes with complications	2
Hemiplegia	2
Moderate or severe kidney disease	2
Tumor (malignant)	2
Leukemia	2
Lymphoma	2
Moderate or severe liver disease	3
Metastatic solid tumor	6
AIDS/HIV	6

Age score: individuals younger than 50 receive 0 points; ages 50–59 receive 1 point; ages 60–69 receive 2 points; ages 70–79 receive 3 points; and those aged 80 and older receive 4 points.

### 2.4 Outcome measures

The primary outcome measures of this study included the incidence of the following in-hospital complications during the hospital stay. **DVT:** Identified through ICD codes and clinical documentation during the hospitalization. **Pressure Ulcers:** Documented cases of pressure ulcers developed during hospitalization, assessed based on nursing and clinical records. **Delirium:** Diagnosed delirium cases, identified through clinical assessments and documented in the medical records, including the use of validated delirium assessment tools when applicable.

### 2.5 Statistical analysis

Statistical analyses were performed using R software version 4.2. Continuous variables were summarized using means and standard deviations SD, while categorical variables were expressed as frequencies and percentages. Baseline characteristics were compared between low-ACCI ≤5 and high-ACCI >5 groups using independent t-tests for continuous variables and chi-square tests for categorical variables. To explore the relationship between ACCI and the risk of in-hospital complications, restricted cubic spline RCS analysis was utilized. This method allowed for the examination of potential nonlinear associations between ACCI scores and the incidence of DVT, pressure ulcers, and delirium. An inflection point was identified to determine the threshold at which risk significantly increased. Multivariate logistic regression analyses were performed to assess the independent effects of ACCI and other covariates on the risk of developing each complication. The model included potential confounders such as age, gender, admission blood parameters hemoglobin concentration, white blood cell count, platelet count, serum albumin levels, Body mass index (BMI), fracture site, operation time, the types of femoral fractures, the surgical procedures performed and the use of low molecular weight heparin (LMWH). Odds ratios (OR) with 95% confidence intervals (95%CI) were calculated to quantify the strength of associations. A *p*-value of <0.05 was considered statistically significant for all analyses.

## 3 Results

### 3.1 Baseline characteristics

The study cohort consisted of 4,134 patients with femoral fractures, stratified into two groups based on the ACCI (Table 2): the low-ACCI group (*N* = 2,510, ACCI ≤ 5) and the high-ACCI group (*N* = 1,624, ACCI > 5). Demographically, the median age of patients in the low-ACCI group was 67 years (range, 21–91), which was significantly younger than that of patients in the high-ACCI group, who had a median age of 78 years (range, 46–91) (*p* < 0.001). Females constituted a majority in both groups, comprising 64.8% of the low-ACCI group and 64.1% of the high-ACCI group, yielding no significant difference (*p* = 0.678). The high-ACCI group exhibited lower mean concentrations of admission hemoglobin and serum albumin compared to the low-ACCI group (*p* < 0.01), reflecting a more compromised physiological state. The BMI was also slightly higher in the low-ACCI group at 26.4 ± 4.7 kg/m^2^, compared to 26.0 ± 4.6 kg/m^2^ in the high-ACCI group, a difference that was statistically significant (*p* = 0.006). In the low-ACCI group, 95.9% of patients received LMWH treatment, while in the high-ACCI group, 97.6% of patients received LMWH treatment, with a statistically significant difference between the two groups (*p* = 0.004). Other variables, such as white blood cell count, platelet count, operation time, fracture type and operation type demonstrated no significant differences between the two cohorts, indicating a homogeneity in these factors across the ACCI stratifications.

**Table 2:** Baseline characteristics of patients with femoral fractures stratified by ACCI score.

Variable	Low-ACCI group (*N* = 2,510)	High-ACCI group (*N* = 1,624)	*p*
Gender (*N*, %)			
Female	1,626 (64.8)	1,041 (64.1)	0.680
Male	884 (35.2)	583 (35.9)	
DVT (*N*, %)			
No	2,276 (90.7)	1,339 (82.5)	<0.001
Yes	234 (9.3)	285 (17.5)	
Pressure ulcer (*N*, %)			
No	2,360 (94.0)	1,409 (86.8)	<0.001
Yes	150 (6.0)	215 (13.2)	
Delirium (*N*, %)			
No	2,271 (90.5)	1,306 (80.4)	<0.001
Yes	239 (9.5)	318 (19.6)	
Age (y, median, range)	67 (21–91)	78 (33–91)	<0.001
Albumin, mean (g/L, mean, SD)	36.3 (5.1)	35.0 (5.9)	<0.001
Hemoglobin (g/L, mean, SD)	105.1 (3.7)	104.8 (3.3)	0.002
Platelet (10^6/L, mean, SD)	231.5 (12.4)	231.5 (10.3)	0.900
White blood cell (*10^9/L, mean, SD)	7.30 (0.64)	7.30 (0.79)	0.782
Fracture type (*N*, %)			
Femoral neck fracture	850 (33.9)	516 (31.8)	0.314
Subtrochanteric fracture	839 (33.4)	573 (35.3)	
Intertrochanteric fracture	821 (32.7)	535 (32.9)	
Operation type (*N*, %)			
Internal fixation	812 (32.4)	546 (33.6)	0.337
Hemiarthroplasty	854 (34.0)	556 (34.2)	
Total hip arthroplasty	834 (33.2)	522 (32.2)	
BMI (kg/m^2^, mean, SD)	26.4 (4.7)	26.0 (4.6)	0.006
Operation time (h)	1.5 (0.3)	1.5 (0.2)	0.078
Low molecular weight heparin (*N*, %)			
Yes	2,406 (95.9)	1,585 (97.6)	0.004
No	104 (4.1)	39 (2.4)	

ACCI, Age-Adjusted Charlson Comorbidity Index; DVT, Deep vein thrombosis; BMI, Body mass index.

### 3.2 Restricted cubic spline analysis

**Figure 2:**
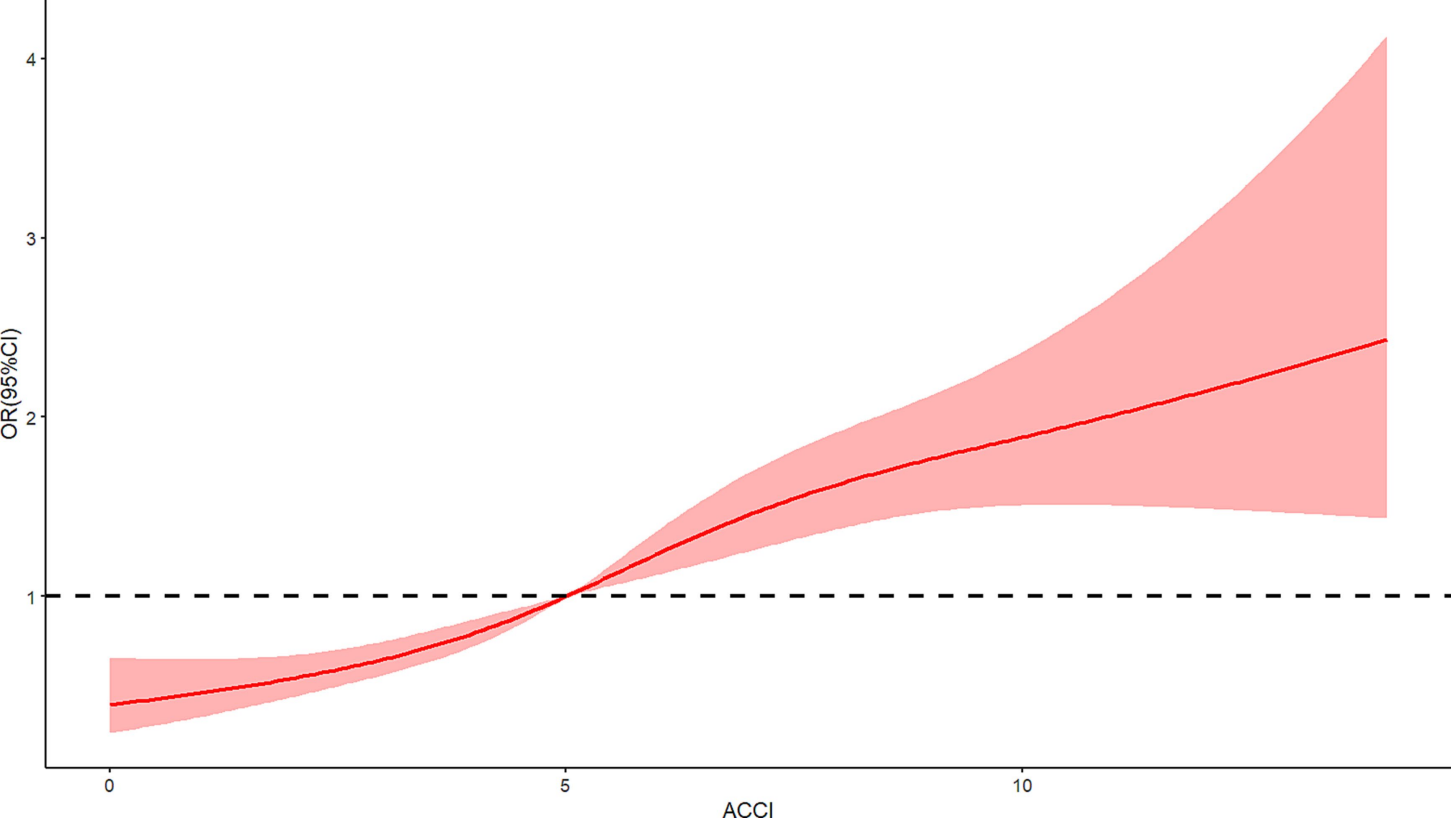
Restricted cubic spline analysis of ACCI and risk of deep venous thrombosis (ACCI, Age-Adjusted Charlson Comorbidity Index).

**Figure 3:**
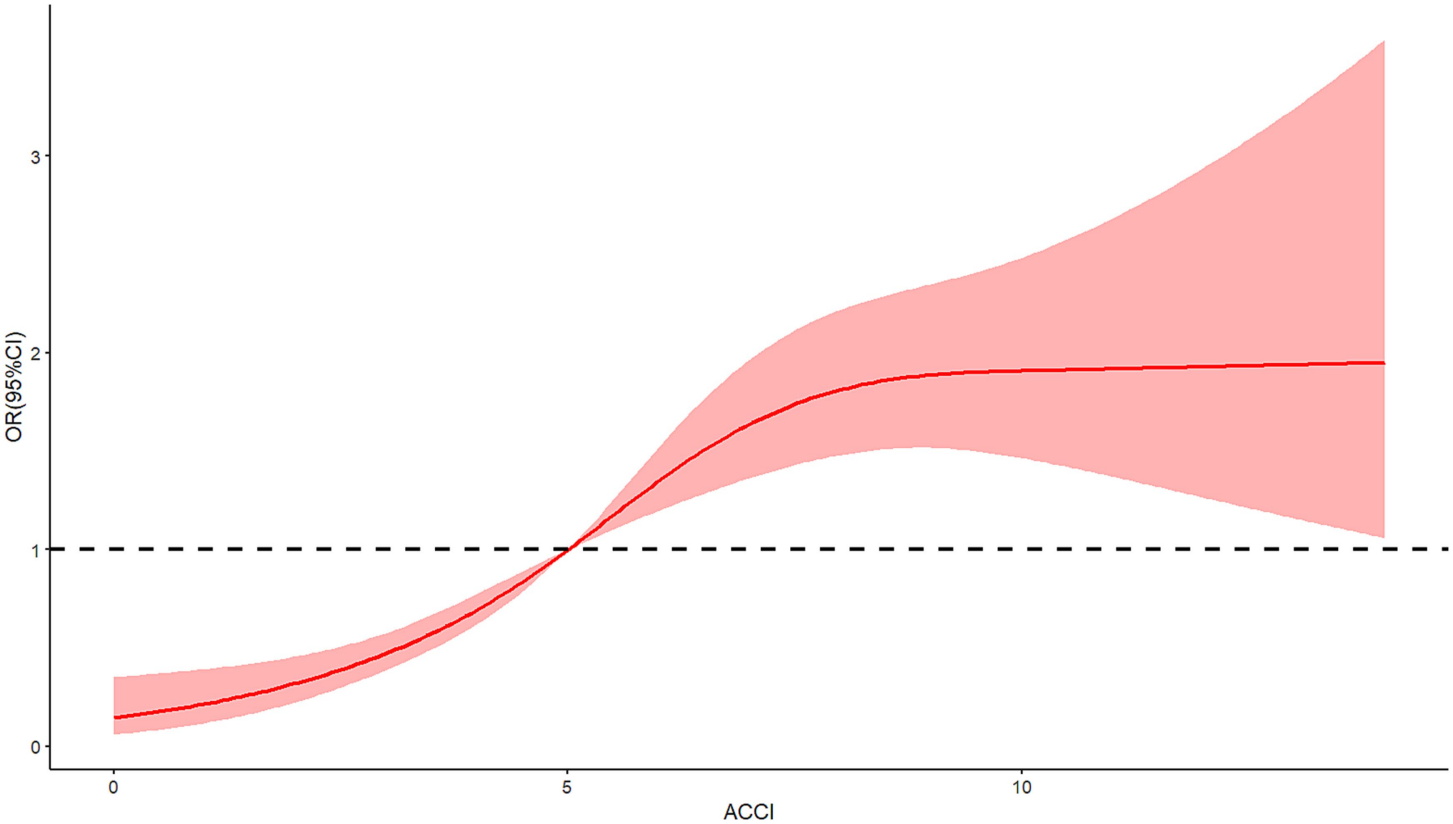
Restricted cubic spline analysis of ACCI and risk of pressure ulcers (ACCI, Age-Adjusted Charlson Comorbidity Index).

**Figure 4:**
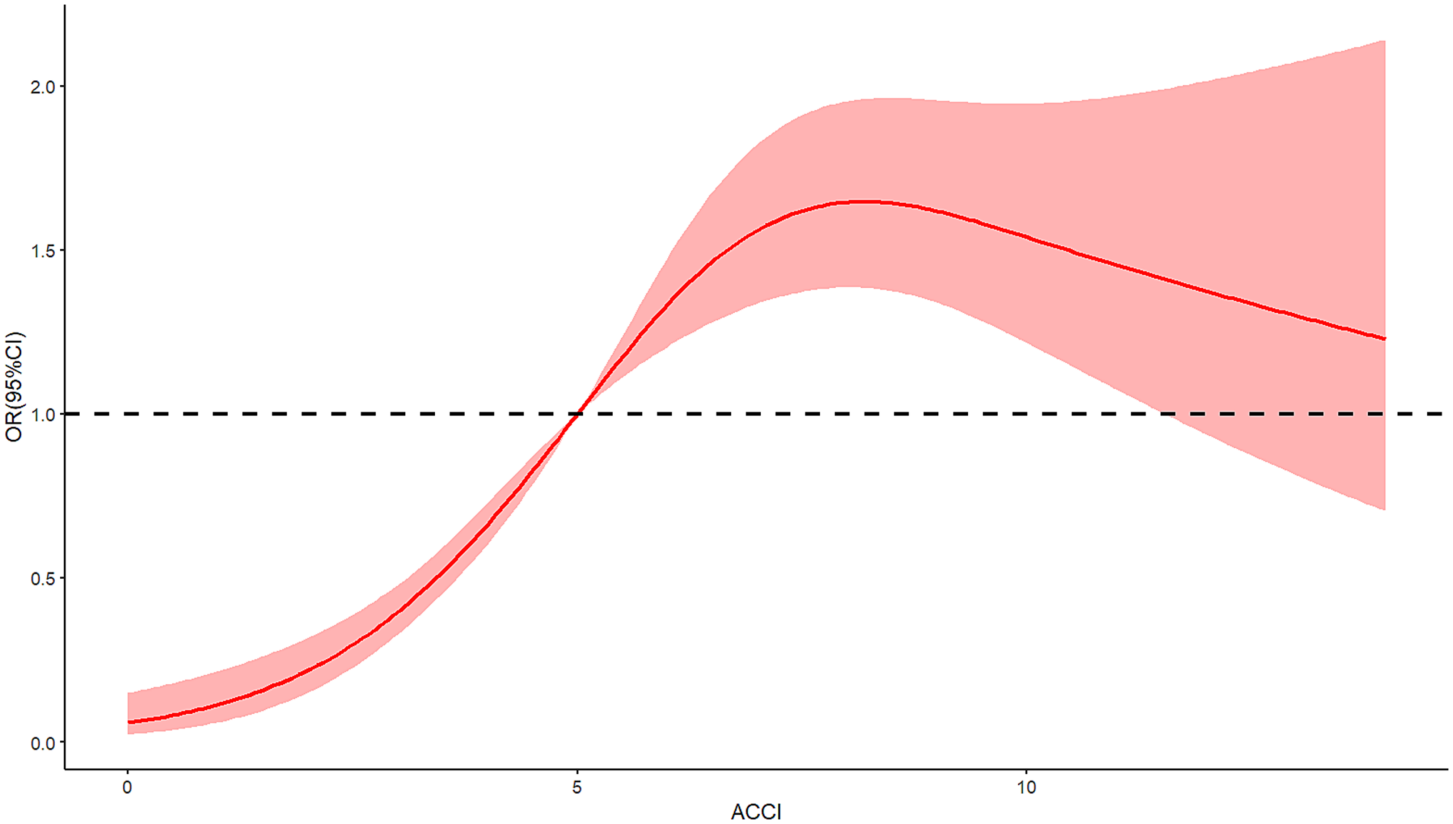
Restricted cubic spline analysis of ACCI and risk of delirium (ACCI, Age-Adjusted Charlson Comorbidity Index).

The relationship between ACCI and in-hospital complications, specifically DVT, pressure ulcers, and delirium, was investigated using restricted cubic spline RCS analysis. This analytical approach revealed a significant nonlinear association between ACCI scores and the risk of each complication. The analysis identified an inflection point at an ACCI score of 5, beyond which the risk of complications markedly increased. For DVT, the spline demonstrated an escalation in risk as ACCI scores rose above 5, indicating a critical threshold for elevated thrombosis risk (*X*^2^ = 7.7, *p* = 0.021, Figure 2). Similar patterns were observed for pressure ulcers (Figure 3) and delirium (Figure 4) with the risk trajectories sharply rising post-ACCI = 5, underscoring the nonlinear dynamics of comorbidity impact on these adverse outcomes. These findings provide quantitative substantiation for using ACCI as a stratification tool in clinical risk assessment for patients with femoral fractures.

### 3.3 Multivariate logistic regression analysis

**Figure 5:**
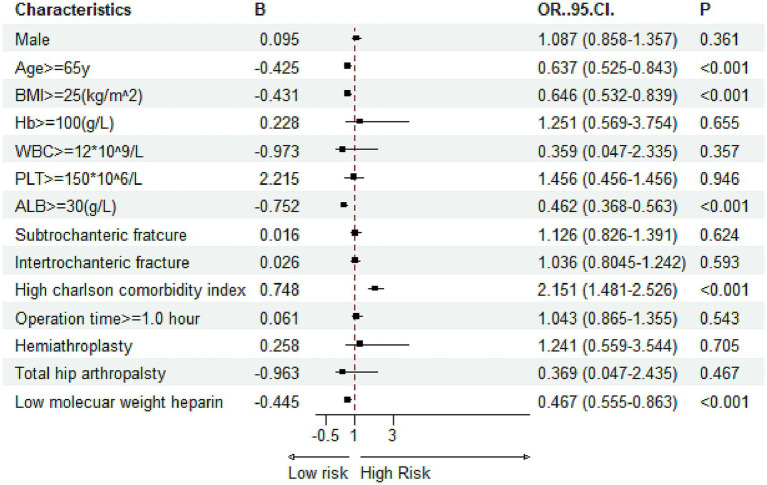
Multivariate logistic regression results for risk factors associated with deep venous thrombosis (Hb, hemoglobin; PLT, platelet; WBC, white blood cell; ALB, albumin; BMI, body mass index).

**Figure 6:**
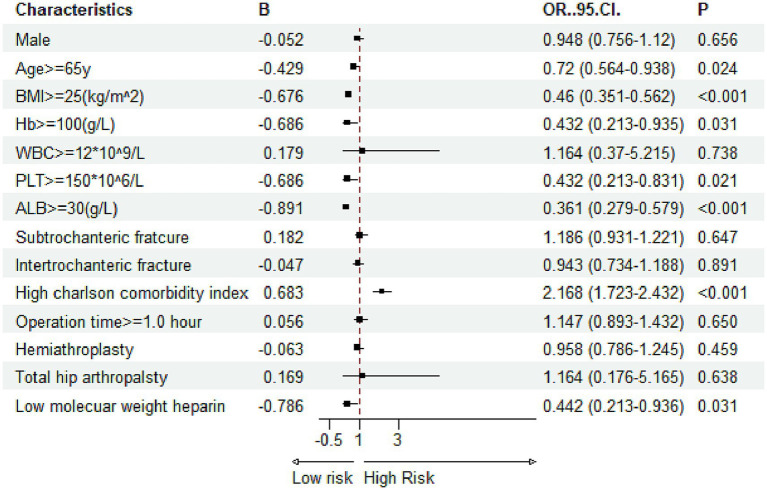
Multivariate logistic regression results for risk factors associated with pressure ulcers (Hb, hemoglobin; PLT, platelet; WBC, white blood cell; ALB, albumin; BMI, body mass index).

**Figure 7:**
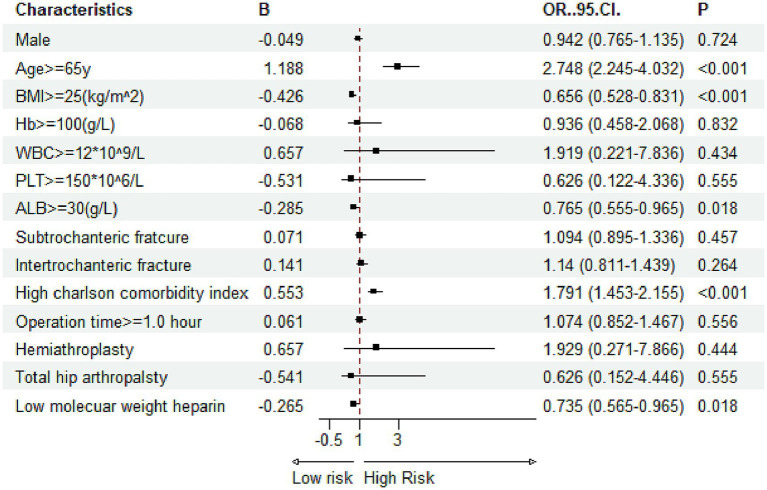
Multivariate logistic regression results for risk factors associated with delirium (Hb, hemoglobin; PLT, platelet; WBC, white blood cell; ALB, albumin; BMI, body mass index).

Multivariate logistic regression was applied to evaluate the independent effects of the ACCI and other covariates on the likelihood of developing in-hospital complications. In the adjusted models, a high ACCI score >5 emerged as a significant independent predictor for all three complications. Specifically, for DVT, patients with a high ACCI had over twice the odds of developing the condition compared to those with a low ACCI (Figure 5, OR = 2.151). For pressure ulcers, the risk was 1.16 times higher in the high-ACCI group (Figure 6, OR = 2.168), and for delirium, the odds were increased by 79.1% (Figure 7, OR = 1.791). Besides ACCI, other independent risk factors identified included lower BMI (<25 kg/m^2^), reduced serum albumin levels (<30 g/L) and not treated with LMWH for DVT, while advanced age, lower hemoglobin levels, compromised albumin levels and not treated with LMWH were also significant for pressure ulcers and delirium. These results highlight the multifactorial nature of complication risk in hospitalized patients with femoral fractures, emphasizing the importance of ACCI in conjunction with other clinical factors for comprehensive risk management.

## 4 Discussion

This study provides compelling evidence of the association between the ACCI and the risk of developing significant in-hospital complications, such as DVT, pressure ulcers, and delirium, among patients with femoral fractures. The data illustrates a pronounced nonlinear relationship with an inflection point at an ACCI score of 5, underscoring the need for targeted clinical strategies for patients exceeding this threshold. The integration of ACCI into routine clinical assessments could substantially enhance patient care by fostering early identification of high-risk individuals. This allows healthcare providers to implement preemptive measures that address specific vulnerabilities associated with high ACCI scores.

### 4.1 Application and clinical significance of the ACCI in elderly patients

The ACCI has significant implications for clinical practice, particularly in the management of elderly patients. Its application extends to various fields, including oncology, cardiology, and surgical care, where understanding a patient’s comorbidity profile is crucial for treatment planning. Research has shown that a higher ACCI score correlates with increased mortality and complications in surgical patients, making it an essential tool for preoperative assessment. For instance, studies have indicated that elderly patients with higher ACCI scores experience worse outcomes following procedures such as coronary artery bypass grafting (10). The ACCI not only aids in identifying high-risk patients who may benefit from more intensive monitoring and tailored interventions but also facilitates informed decision-making regarding the appropriateness of surgical procedures. Moreover, the ACCI can guide postoperative care strategies and resource allocation, ensuring that healthcare providers can deliver optimal care tailored to the unique needs of older adults. Thus, the ACCI serves as a vital component in enhancing patient safety and improving clinical outcomes in the geriatric population (11).

### 4.2 Clinical features and risk factors of femoral fractures

Femoral fractures, particularly in the elderly population, present a significant clinical challenge due to their associated morbidity and mortality. The clinical features of femoral fractures can vary widely, influenced by the patient’s age, comorbidities, and the mechanism of injury. In older adults, these fractures often result from low-energy falls, reflecting the fragility of their bones due to conditions such as osteoporosis. The types of femoral fractures commonly encountered include femoral neck fractures, intertrochanteric fractures, and subtrochanteric fractures, each with distinct implications for treatment and recovery. For instance, femoral neck fractures are particularly concerning as they are associated with a higher risk of avascular necrosis and nonunion, necessitating careful surgical intervention and rehabilitation strategies (12). Furthermore, the clinical outcomes following these fractures can be severely impacted by factors such as delayed surgical intervention, with studies showing that surgical delays exceeding 48 h significantly increase mortality rates (13). Understanding these clinical features is crucial for developing effective management protocols for elderly patients suffering from femoral fractures.

### 4.3 Comorbid factors affecting femoral fracture healing

The healing process following femoral fractures in elderly patients is significantly influenced by various comorbid factors. Conditions such as osteoporosis, diabetes, and cardiovascular diseases can impair bone healing and increase the risk of complications. For instance, osteoporosis is a major risk factor for both the occurrence of femoral fractures and the subsequent healing process, as it leads to decreased bone density and strength (14). Similarly, diabetes has been associated with delayed healing and an increased risk of infections post-surgery, which can further complicate recovery (15). Other comorbidities, such as chronic kidney disease and obesity, also play a critical role in the healing process, with studies indicating that patients with these conditions may experience higher rates of nonunion and complications (16). Additionally, the presence of multiple comorbidities can lead to a more complex clinical picture, necessitating a multidisciplinary approach to management that addresses not only the fracture itself but also the underlying health issues that may impede recovery (13). Understanding these comorbid factors is essential for optimizing treatment strategies and improving outcomes for elderly patients with femoral fractures.

### 4.4 Correlation studies between ACCI and DVT incidence

The association between the ACCI and the incidence of DVT has been a subject of extensive research. ACCI is a scoring system that evaluates the severity of acute clinical conditions and their potential to contribute to adverse outcomes, including thromboembolic events. Studies have shown that higher ACCI scores are associated with an increased risk of DVT, particularly in hospitalized patients or those undergoing surgical procedures (17). This correlation is likely due to the cumulative effect of multiple acute conditions that can lead to venous stasis, endothelial injury, and hypercoagulability. For instance, patients with severe infections, trauma, or malignancies often present with elevated ACCI scores, which reflect their heightened risk for DVT (18). Moreover, the use of ACCI as a predictive tool in clinical settings can aid healthcare providers in identifying high-risk patients who may benefit from preventive measures, such as anticoagulation therapy or mechanical prophylaxis. Future research is needed to validate the effectiveness of ACCI in diverse patient populations and to explore its integration into existing risk assessment models for DVT.

### 4.5 The mechanism of pressure ulcer formation and prevention strategies

Pressure ulcers, also known as bedsores or decubitus ulcers, are localized injuries to the skin and/or underlying tissue, typically over bony prominences, due to prolonged pressure, often in combination with shear and friction. The mechanisms underlying pressure ulcer formation are multifactorial, involving external pressure, tissue perfusion, and the individual’s health status. When pressure is exerted on the skin, it impedes blood flow to the area, leading to ischemia and cell death. Factors such as moisture, friction, and shear forces can exacerbate tissue damage, particularly in vulnerable populations such as the elderly or those with limited mobility. The classification of pressure ulcers, based on the National Pressure Injury Advisory Panel (NPIAP) guidelines (19), ranges from Stage I (non-blanchable erythema) to Stage IV (full-thickness tissue loss with exposed bone, tendon, or muscle). The impact of pressure ulcers on patients is profound, leading to pain, increased risk of infection, longer hospital stays, and decreased quality of life. Moreover, the financial burden on healthcare systems is significant, with estimates suggesting that the treatment of pressure ulcers can cost thousands of dollars per patient, highlighting the necessity for effective prevention strategies (20).

### 4.6 Classification of pressure ulcers and their impact on patients

The classification of pressure ulcers is critical for understanding their severity and guiding treatment. According to the NPIAP, pressure ulcers are classified into four stages: Stage I involves intact skin with non-blanchable redness, Stage II presents as a partial-thickness loss of skin, Stage III is characterized by full-thickness tissue loss without exposed bone, and Stage IV indicates full-thickness tissue loss with exposed bone, muscle, or tendon. Each stage has implications for patient care, as deeper ulcers often require more complex management strategies. The impact of pressure ulcers extends beyond physical discomfort; they can lead to significant psychological distress and social isolation. Patients with pressure ulcers often experience increased pain and discomfort, which can lead to anxiety and depression. Furthermore, the presence of pressure ulcers can complicate existing medical conditions, prolong hospitalization, and increase the risk of mortality. Studies have shown that patients with advanced pressure ulcers have a higher incidence of infections and comorbidities, which can further deteriorate their overall health status. Therefore, understanding the classification and implications of pressure ulcers is essential for healthcare providers in order to implement timely and effective interventions to mitigate their impact on patient wellbeing (21).

### 4.7 Application of ACCI in pressure ulcer risk assessment

The Application of the Acute Care for Elders (ACE) Clinical Initiative (ACCI) in assessing the risk of pressure ulcers is a significant advancement in preventive care. The ACCI framework emphasizes a comprehensive approach to patient assessment, focusing on the unique needs of elderly patients who are at higher risk for pressure ulcer development due to factors such as immobility, poor nutrition, and comorbidities. The ACCI utilizes a multidimensional assessment tool that evaluates various risk factors, including mobility, sensory perception, moisture, activity level, and nutrition, to stratify patients based on their risk for developing pressure ulcers. By implementing this structured risk assessment, healthcare providers can identify high-risk patients early and initiate preventative measures, such as repositioning schedules, nutritional support, and skin care protocols. Moreover, integrating the ACCI into clinical practice not only enhances patient outcomes but also promotes a culture of safety and quality improvement within healthcare settings. As such, the ACCI serves as a valuable resource in the ongoing efforts to prevent pressure ulcers and improve the overall quality of care for vulnerable patient populations (22).

### 4.8 The etiology and management of delirium

Delirium is a complex neuropsychiatric syndrome characterized by an acute disturbance in attention and cognition, often fluctuating in severity (23, 24). It is particularly prevalent among elderly patients, especially those undergoing surgical procedures or experiencing acute medical illnesses. The underlying mechanisms of delirium are multifactorial, often involving a combination of metabolic disturbances, infections, medications, and environmental factors. The identification and management of delirium are crucial, as it is associated with increased morbidity, prolonged hospital stays, and higher mortality rates (25–27). Effective management strategies include non-pharmacological interventions such as optimizing the environment, ensuring adequate hydration and nutrition, and employing cognitive stimulation techniques. Pharmacological treatments may be necessary in some cases, particularly when patients exhibit severe agitation or distress, but should be approached with caution due to the potential for adverse effects in vulnerable populations (28, 29).

### 4.9 The correlation between ACCI and the incidence of delirium

The ACCI has emerged as a significant predictor of postoperative delirium (POD) in elderly patients. The ACCI accounts for the number and severity of comorbid conditions, which can exacerbate the risk of developing delirium (30, 31). For instance, a prospective observational study indicated that patients with a higher ACCI were more likely to experience POD, with the index serving as an independent risk factor even after adjusting for other variables such as age and preoperative cognitive status (32). The predictive value of the ACCI in assessing the risk of delirium underscores the importance of comprehensive preoperative evaluations that include assessing comorbidities. This approach can facilitate targeted interventions to reduce the risk of delirium in at-risk populations (11, 33).

### 4.10 Practical recommendations for ACCI application

Incorporating insights from our study into clinical practice can greatly enhance the care of elderly patients with femoral fractures. We recommend utilizing the ACCI for preoperative risk stratification to identify high-risk patients, guiding tailored assessments and surgical decisions. Early postoperative anticoagulation with low molecular weight heparin is also advised to reduce the risk of DVT. Educating patients and their families about their comorbidities will encourage active participation in their care. Moreover, a multidisciplinary team approach involving orthopedic surgeons, geriatricians, physiotherapists, and nurses is crucial for addressing the diverse needs of these patients. By implementing these strategies, we aim to improve patient outcomes and optimize management for elderly individuals at risk of postoperative complications.

### 4.11 Strengths and limitations

This study benefits from utilizing a large and well-defined cohort from the MIMIC-IV database, enhancing the robustness and generalizability of our findings regarding the relationship between the Age-Adjusted Charlson Comorbidity Index (ACCI) and in-hospital complications among elderly patients with femoral fractures. The methodological rigor, including multivariate analysis, strengthens our conclusions and provides valuable insights for risk stratification in clinical practice. However, the retrospective nature of the analysis limits the establishment of causal relationships, and the single-institution data may restrict the generalizability of the results. Potential data gaps and reliance on electronic health records may not fully capture the patient experience, especially for subclinical complications. To address these limitations, future research should incorporate multicenter data and prospective studies while integrating patient-reported outcomes for a more comprehensive understanding of risks in managing patients with femoral fractures.

## 5 Conclusion

This study reinforces the predictive capacity of the Age-Adjusted Charlson Comorbidity Index for assessing the risk of critical in-hospital complications among femoral fracture patients. Employing ACCI in clinical practice provides healthcare professionals with a valuable tool to stratify patient risk and personalize care interventions, leading to enhanced patient outcomes and reduced healthcare burdens.

## Data Availability

The raw data supporting the conclusions of this article will be made available by the authors without undue reservation.
